# Identification of epigenetic variation associated with synchronous pod maturity in mungbean (*Vigna radiata* L.)

**DOI:** 10.1038/s41598-020-74520-z

**Published:** 2020-10-15

**Authors:** Jungmin Ha, Hakyung Kwon, Kang-Heum Cho, Min Young Yoon, Moon Young Kim, Suk-Ha Lee

**Affiliations:** 1 Department of Agriculture, Forestry and Bioresources, Seoul National University, Seoul, 08826 Republic of Korea; 2 Department of Plant Science, Gangneung-Wonju National University, Gangneung, Republic of Korea; 3 Plant Genomics and Breeding Institute, Seoul National University, Seoul, 08826 Republic of Korea

**Keywords:** Plant breeding, Epigenomics

## Abstract

Cytosine methylation in genomic DNA affects gene expression, potentially causing phenotypic variation. Mungbean, an agronomically and nutritionally important legume species, is characterized by nonsynchronous pod maturity, resulting in multiple harvest which costs extra time and labor. To elucidate the epigenetic influences on synchronous pod maturity (SPM) in mungbean, we determined the genome-wide DNA methylation profiles of eight mungbean recombinant inbred lines (RILs) and their parental genotypes, and compared DNA methylation profiles between high SPM and low SPM RILs, thus revealing differentially methylated regions (DMRs). A total of 3, 18, and 28 pure DMRs, defined as regions showing no significant correlation between nucleotide sequence variation and methylation level, were identified in CpG, CHG, and CHH contexts, respectively. These DMRs were proximal to 20 genes. Among the 544 single nucleotide polymorphisms identified near the 20 genes, only one caused critical change in gene expression by early termination. Analysis of these genome-wide DNA methylation profiles suggests that epigenetic changes can influence the expression of proximal genes, regardless of nucleotide sequence variation, and that SPM is mediated through gibberellin-mediated hormone signaling pathways. These results provide insights into how epialleles contribute to phenotypic variation and improve SPM in mungbean cultivars.

## Introduction

Mungbean (*Vigna radiata* L.) is a grain legume crop widely cultivated in Asia as an important source of nutrients such as proteins, amino acids, folate, and iron^1^. Because mungbean plants are fast-growing and can fix atmospheric nitrogen through symbiosis with *Rhizobium*, cultivating mungbean in rotation with other grain crops increases the yield of the subsequent crop and decreases the incidence of pests^2^. Despite the agronomic and nutritional importance of mungbean, desirable agronomic traits such as determinate growth habit, simultaneous flowering, and synchronous pod maturity (SPM) have not been thoroughly achieved in modern breeding programs.

Mungbean exhibits nonsynchronous pod maturity^3^. If mungbean is harvested only once during the entire growing season (at the R6 growth stage, when most pods reach maturity), approximately 50% of the yield potential is lost because mungbean plants continue flowering and producing pods^4^. Delayed harvest also causes substantial yield losses because mungbean plants are more susceptible to pests and pathogens at later growth stages, and mature and dried pods are more likely to shatter. Therefore, a single mungbean crop must be harvested multiple times to reduce yield loss. Moreover, each harvest must be conducted carefully to avoid any damage to plants, which makes mechanical harvesting inefficient. Although the genome of mungbean has been sequenced^5^, the genetic or epigenetic basis of SPM remains unknown because SPM is a complex trait affected by multiple factors and few quantitative trait locus (QTL) mapping or gene cloning studies have been conducted in mungbean.

Phenotypic variation is determined by the plant genotype, environment, and genotype × environment interaction. DNA methylation, a heritable epigenetic modification, also contributes to phenotypic variation^6^. Epialleles are classified into three major groups, based on their dependence on the genotype: obligate epialleles, which are completely dependent on genetic variation; pure epialleles, which are independent of any genetic variation; and facilitated epialleles, which originate from genetic variants but do not necessarily depend on genetic variation for maintenance^7^. However, the effect of epigenetic variation on the phenotype remains unclear because only a few epigenetic marks have been characterized to date^8^. In soybean (*Glycine max* L.), the model species closest to mungbean, carbohydrate metabolism pathway genes are enriched in epigenetic variation^9^. In tomato (*Solanum lycopersicum* L.) fruits, vitamin E content is determined by epialleles^10^. Although genome-wide DNA methylation patterns have been investigated in two mungbean genotypes, VC1973A and V2984, epigenetic factors associated with any agronomic trait have not been studied in mungbean^5,11^.

In this study, we aimed to characterize the epigenetic variation associated with SPM in mungbean. Whole genome resequencing and bisulfite-sequencing of four high SPM recombinant inbred lines (RILs), four low SPM RILs, and two parental genotypes (VC1973A and V2984) were conducted to determine genome-wide DNA methylation patterns. The mungbean cultivar VC1973A is widely grown in Asia and exhibits high SPM, whereas V2984 is a Korean landrace with low SPM^3^. Comparison of these DNA methylation profiles between high vs. low SPM lines revealed pure differentially methylated regions (DMRs), independent of genetic variation. Genes in these DMRs showed differential expression levels between the parental genotypes VC1973A and V2984. These results advance our understanding of SPM in mungbean and of the contribution of epialleles to phenotypic variation.

## Results

### Variation in the SPM index of the RIL population

**Figure 1:**
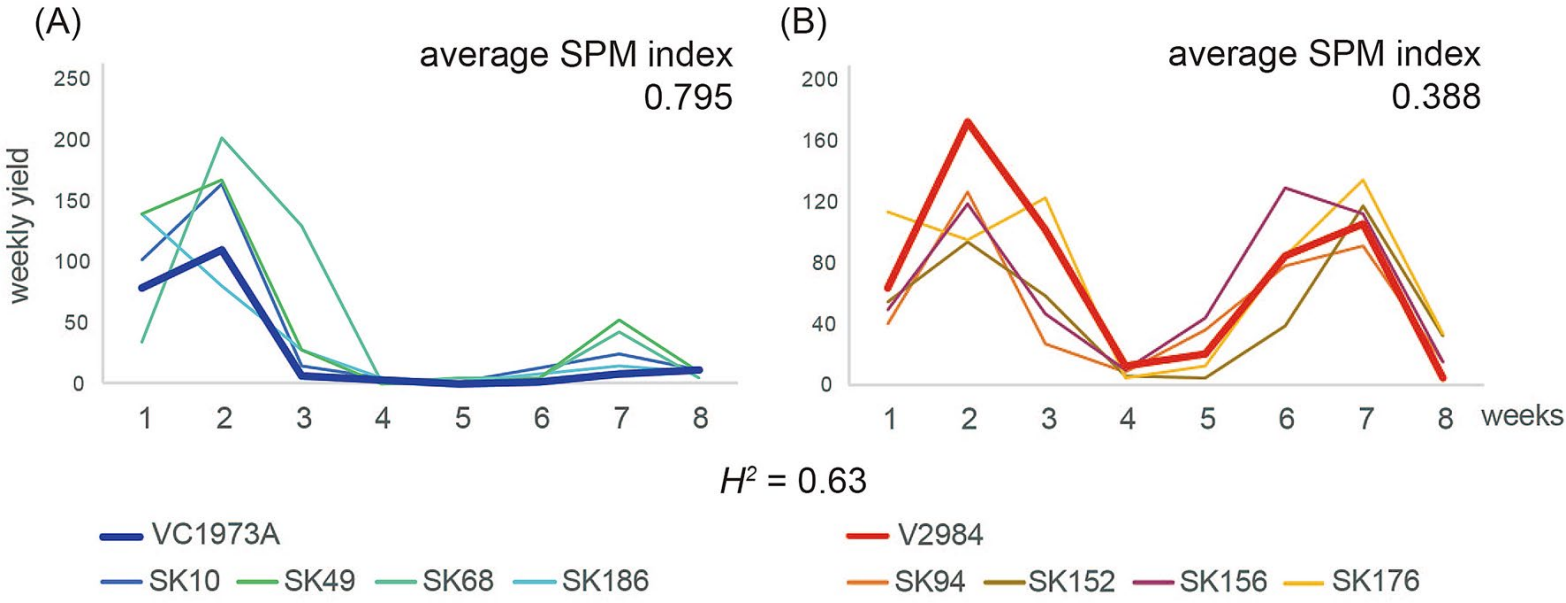
Weekly harvest for SPM index calculation. Ten plants per line were harvested weekly. X and Y axes indicate weeks and pod numbers weekly harvested, respectively. A value of SPM close to 1 theoretically represents that all pods matured at the same time. (**A**) VC1973A and four lines that show the highest SPM. (**B**) V2984 with four lines that show the lowest SPM^3^.

A total of 187 RILs were harvested weekly for 8 weeks (Figure S1). The SPM index (range: 0 to 1) was calculated for each RIL. A value of SPM close to 1 theoretically indicates that all pods mature at the same time. The number of pods in VC1973A (maternal parent) was the highest at week 2 (SPM index: 0.862), while the number of pods in V2984 (paternal parent) peaked again at week 7 (SPM index: 0.487) (Fig. 1, Table S1)^3^. Among the 187 RILs, we selected the four RILs (SK10, SK68, SK186, and SK49) with the highest SPM index (0.792, 0.783, 0.772, and 0.764, respectively) and the four RILs (SK94, SK152, SK176, and SK156) with the lowest SPM index (0.394, 0.374, 0.364, and 0.319, respectively) for bisulfite-sequencing and resequencing analyses with their parental lines. Groups of RILs with high and low SPM indices showed similar patterns of weekly harvests as VC1973A and V2984, respectively, indicating that the group with a high SPM index showed higher synchronicity in pod maturity (Fig. 1).

### *Identification of DMRs associated with SPM *via* bisulfite-sequencing*

To identify candidate epialleles involved in the regulation of SPM, the selected ten lines, including eight RILs and two parental genotypes, were subjected to Methyl-Seq after bisulfite conversion (Table S2). The bisulfite conversion efficiencies ranged from 98.5 to 99.0%, which were sufficiently high for subsequent biological applications such as Methyl-Seq analysis, as reported previously^12^. A total of 488, 336, and 406 DMRs in contexts of CHG, CHH, and CpG methylation were identified (Figure S2), of which 134 (36%), 160 (19%), and 65 (33%) were located at genic regions, respectively, including upstream sequence (− 2 kb from the transcription start site), 5′ untranslated region (5′-UTR), coding sequence (CDS), intron, 3′-UTR, and downstream sequence (+ 2 kb from the transcription termination site) (Table 1). After the removal of genes redundant in DMRs of all methylation contexts, 211 genes were identified as proximal to DMRs. Among the genes in genic DMRs associated with SPM, gene ontology (GO) analysis revealed the enrichment of reproductive structure development and transcription factor activity-related GO terms such as ‘nucleotide binding’ and ‘catalytic activity’ (Figure S3). Additionally, ‘metabolic pathways’, ‘biosynthesis of secondary metabolites’, and ‘plant hormone signal transduction’ were the top three enriched pathways in the KEGG database (Table S3)^13^.

**Table 1:** The numbers of DMRs.

Methylation type		CpG	CHG	CHH
The number of DMRs		406	448	336
Intergenic		272	288	271
Genic	Upstream (2kbp)	50	40	25
	5′-UTR	6	6	1
	CDS	69	83	7
	Intron	67	98	21
	3′-UTR	6	9	1
	Downstream (2kbp)	43	47	23

### Methylation levels at genic DMRs

**Figure 2:**
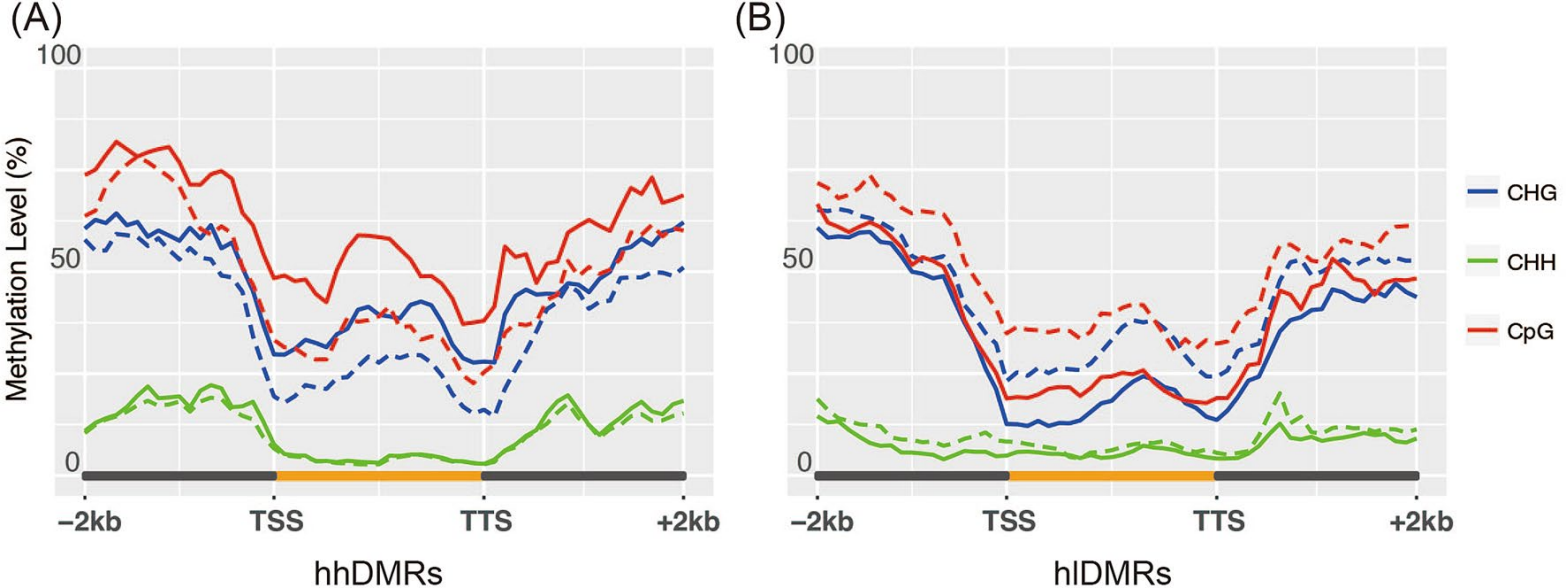
Average methylation levels of cytosines on genic DMRs (**A**) where methylation level is significantly higher in high SPM group than low SPM group (hhDMRs) and (**B**) where methylation level is significantly higher in low SPM group than high SPM group (hlDMRs). Solid and dotted lines indicate methylation levels of DMRs in high and low SPM group, respectively. Upstream region, gene body and downstream region consist of 20 bins and average methylation levels are calculated over three sliding windows.

The distribution of methylation at genic regions, including upstream and downstream sequences of genes in mungbean, was consistent with that in Arabidopsis and soybean (Fig. 2)^14,15^. A slight increase in methylation was detected in the middle of the gene body, and a steep increase was observed in upstream and downstream sequences in mungbean. On the basis of differences in methylation levels between high and low SPM groups, we classified genic DMRs into two sets: DMRs with significantly higher methylation in the high SPM group than in the low SPM group (hhDMRs), and DMRs with significantly higher methylation in the low SPM group than in the high SPM group (hlDMRs). In both DMR groups, differences in average methylation levels for CpG and CHG between high and low SPM lines were higher in gene bodies than in upstream and downstream regions. Cytosine residues methylated in the CpG context showed the largest difference in methylation levels in genic DMRs, whereas those methylated in the CHH context showed the lowest difference in methylation levels (Table S4). This result was consistent with the previous observation that methylation level of CHH had the least effect on gene expression levels in mungbean^11^.

### Genetic diversity and pure genic DMRs

**Figure 3:**
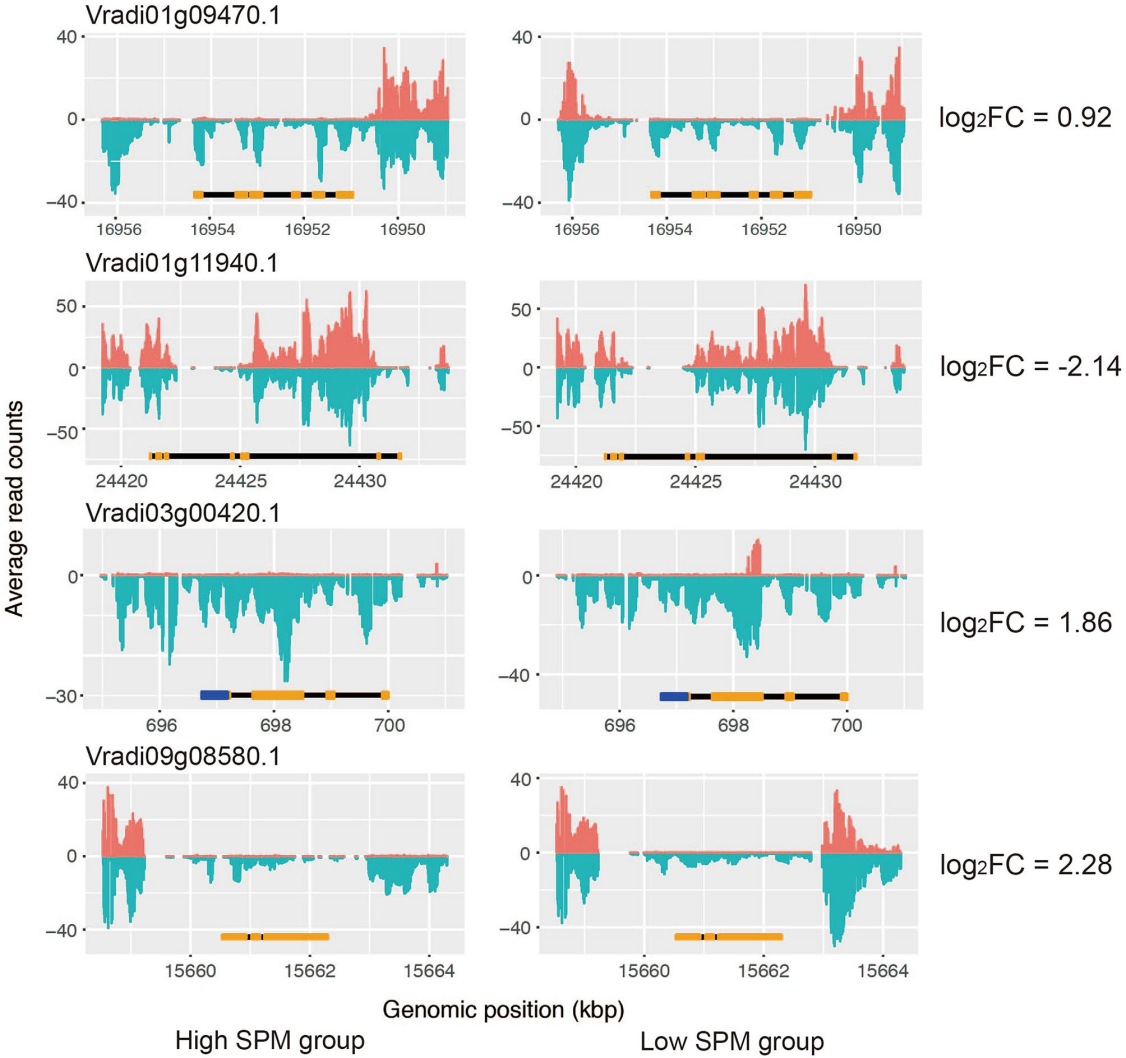
Comparison of average methylation levels of four specific genes between high and low SPM RIL groups. The average number of methylated and unmethylated cytosines of the genes proximal to pure genic DMRs are indicated as red and green, respectively. Left and right columns represent high and low SPM groups, respectively. Yellow, black and blue bars represent exon, intron and UTR. Negative log_2_FC indicates higher expression in V2984.

To identify pure DMRs, where no SNPs are significantly associated with methylation levels, approximately 5 Gb of Illumina raw data were generated for each RIL (Table S5). Raw sequence reads of eight RILs and V2984 (paternal genotype) were mapped against the mungbean reference genome (VC1973A; maternal genotype)^5^. The number of SNPs and Indels in each line ranged from 269 to 798 k and 26 to 134 k, respectively. Out of 282,517 SNPs identified among the ten genotypes, 40,178 SNPs co-segregated with high and low SPM groups. A total of 1081, 1343, and 397 SNPs were located within ± 1 kb of the DMRs in CpG, CHG, and CHH contexts, respectively; among these, 1059, 1248, and 247 SNPs, respectively, were significantly associated with methylation levels of the DMRs (*p* < 0.01) (Table 2, Table S6). Thus, three CpG, 18 CHG, and 28 CHH pure DMRs were revealed, of which one CpG, ten CHG, and ten CHH were pure genic DMRs located within ± 2 kb sequence of 20 genes. These genes included Vradi11g00660 (log_2_fold-change [log_2_FC] = 4.51; encodes an expressed protein), Vradi09g08580 (log_2_FC = 2.28; encodes a receptor-like protein), Vradi01g11940 (log_2_FC = − 2.14; encodes a peroxisomal membrane protein), Vradi03g00420 (log_2_FC = 1.86; encodes a gibberellin receptor GID1L2), Vradi08g18940 (log_2_FC = 0.94; encodes an F-box domain containing protein), and Vradi01g09470 (log_2_FC = 0.92; encodes a transmembrane protein) (Table 3, Fig. 3)^11^. The difference in methylation status possibly affected the difference in expression levels of these genes between VC1973A and V2984, independent of nucleotide sequence variation.

**Table 2:** The number of SNPs within DMRs.

	No. of SNPs within genic DMRs	No. of SNPs significantly associated with methylation levels	No. of DMRs with no significant SNPs	No. of genic DMRs with no significant SNPs
CpG	1081	1059	3	1
CHG	1343	1248	18	10
CHH	397	247	28	10

The association between SNPs and methylation levels was tested by independent-two-sample t-test with *p*-value < 0.01.

**Table 3:** List of genes in pure genic DMRs.

	Chr	Start	End	Position	Gene name	Log_2_FC	*At* orthologs	Description
CpG	Vr04	3,931,482	3,931,659	CDS	Vradi04g01340	− 0.25	AT4G13050.1	Acyl-ACP thioesterase, putative, expressed
CHG	Vr01	16,955,933	16,956,106	Upstream	Vradi01g09470	0.92	AT3G26950.1	Transmembrane protein
	Vr01	17,047,495	17,048,407	Intron	Vradi01g09510	0.10	AT5G16040.1	Regulator of chromosome condensation domain containing protein, expressed
	Vr02	20,756,126	20,757,336	Intron	Vradi02g11080	0.19	AT5G54310.1	GTPase-activating protein, putative, expressed
	Vr03	698,270	698,471	CDS	Vradi03g00420	1.86	AT3G63010.1	Gibberellin receptor GID1L2, putative, expressed
	Vr04	3,931,549	3,931,660	CDS	Vradi04g01340	− 0.25	AT4G13050.1	Acyl-ACP thioesterase, putative, expressed
	Vr06	18,539,658	18,541,211	Upstream	Vradi06g09130	0.00	AT2G20030.1	Zinc finger, C3HC4 type domain containing protein, expressed
	Vr07	3,203,768	3,204,077	Intron	Vradi07g01900	0.74	AT4G16444.1	Expressed protein
	Vr09	15,662,981	15,663,298	Downstream	Vradi09g08580	2.28	AT4G08850.1	Receptor-like protein kinase precursor, putative, expressed
	Vr09	15,663,317	15,663,764	Downstream	Vradi09g08580			
	Vr11	666,942	667,161	Downstream	Vradi11g00660	4.51	AT5G16810.1	Expressed protein
				Downstream	Vradi11g00670	0.00	AT1G05370.1	SEC14 cytosolic factor family protein, putative, expressed
CHH	Vr01	24,430,241	24,430,310	Intron	Vradi01g11940	− 2.14	AT5G19750.1	Mpv17 / PMP22 family domain containing protein, expressed
	Vr01	24,895,975	24,896,037	Downstream	Vradi01g12060	0.00	AT3G55270.1	Dual specificity protein phosphatase, putative, expressed
	Vr04	3,931,562	3,931,663	CDS	Vradi04g01340	− 0.25	AT4G13050.1	Acyl-ACP thioesterase, putative, expressed
	Vr06	11,121,283	11,121,365	Intron	Vradi06g07570	− 0.07	AT5G05200.1	ABC1 family domain containing protein, putative, expressed
	Vr06	28,854,603	28,854,642	Upstream	Vradi06g11970	0.41	AT5G36210.1	OsPOP12—Putative Prolyl Oligopeptidase homologue, expressed
	Vr06	29,589,158	29,589,230	CDS	Vradi06g12210	0.30	AT5G10690.1	CBS domain containing protein, expressed
	Vr07	46,917,546	46,917,654	Upstream	Vradi07g23770	− 0.19	AT1G20200.1	26S proteasome non-ATPase regulatory subunit 3, putative, expressed
	Vr08	35,975,649	35,975,709	Intron	Vradi08g15730	0.73	AT3G11910.2	Ubiquitin carboxyl-terminal hydrolase, putative, expressed
	Vr08	40,787,891	40,787,991	Upstream	Vradi08g18940	0.94	AT3G48880.2	OsFBX389—F-box domain containing protein, expressed
	Vr08	9,299,650	9,299,676	Upstream	Vradi08g04840	0.00	AT3G56960.1	Phosphatidylinositol-4-phosphate 5-kinase, putative, expressed
				CDS	Vradi08g04830	0.00	AT5G65700.2	Receptor protein kinase CLAVATA1 precursor, putative, expressed

Log_2_FC indicates the differences of the expression levels between VC1973A and V2984. Negative value for Log_2_FC indicates higher expression in V2984.

## Discussion

There is an increased interest in utilizing epialleles as a potential breeding resource for harnessing previously unassessed heritable variation affecting plant phenotypes^15^. Because differential DNA methylation, together with genetic variation, can affect gene expression and thus phenotypic variation, DNA methylation should be considered as a critical factor in molecular breeding^6,14^.

To measure SPM in mungbean, we had tried three different approaches. The first approach was the length of productive days indicating each plant produces at least three pods when harvested each week. Synchronous plants should have shorter productive day compared to non-synchronous plants. The second approach was the ratio between the highest pod number among weekly harvests and total pod number. Synchronous plant should have a value close to one. These two approaches did not represent SPM well because of the presence of the second peak of weekly harvest (Fig. 1). The third approach was SPM index used in this study, which showed synchronicity of pod maturity the best among three approaches.

To elucidate the epigenetic effect on SPM in mungbean, DMRs were identified between high and low SPM groups, and genic DMRs were subsequently selected for bisulfite-sequencing and resequencing analyses (Table 1). Transcription factor activity-related GO terms were enriched among the 211 genes proximal to SPM-associated genic DMRs, and 17 of these genes encoded transcription factors (Figure S3, Table S7). Because ‘plant hormone signal transduction’ was one of the enriched KEGG pathways in our data (Table S3), three genes, including Vradi07g03990, Vradi08g06860, and Vradi0284s00060 encoding bZIP, AP2, and ARF transcription factors, respectively, were mapped to auxin, abscisic acid, and ethylene-related signal transduction pathways, respectively, suggesting that these plant hormones participate in the regulation of SPM in mungbean (Figure S4)^13^.

To investigate whether gene expression was affected by nucleotide sequence variation or not, we identified 544 SNPs within 2 kb upstream and downstream of 20 genes proximal to 21 pure DMRs (Table 3). These DMRs were located in upstream, CDS, intron, and downstream regions of the 20 genes. The number of SNPs in each gene varied from 1 to 100 (Figure S5). Most SNPs (> 80%) were located in non-coding regions of genes (Table S6). Only 24 SNPs were missense variants, and one SNP resulted in a premature stop codon, indicating that most nucleotide variants around the genes do not have a significant impact on the expression level of the 20 genes.

**Figure 4:**
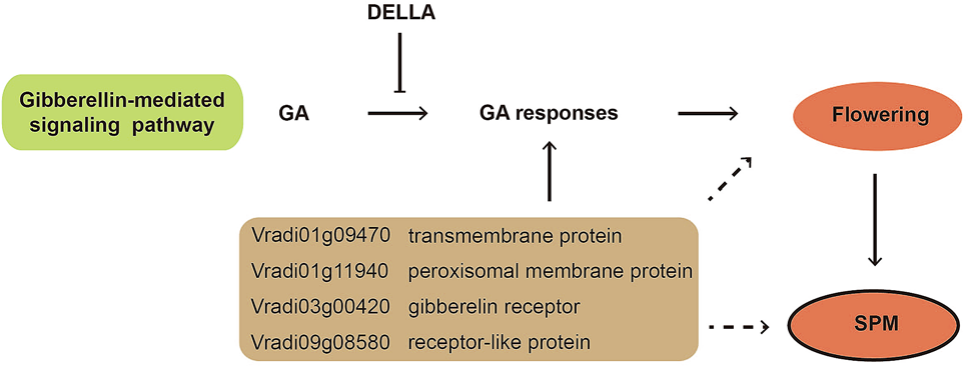
Gibberellin mediated flowering pathway. Plants flower in response to gibberellic acid and DELLA proteins negatively regulate this pathway. When DELLA proteins are degraded, the pathway is turned on. GA indicates gibberellic acids.

Flowering pathways have been well studied in model plant species: photoperiod, autonomous, vernalization and gibberellin pathways^16^. Gibberellins are plant hormones that regulate various developmental processes, including flower induction and development, seed development, and fruit senescence^17^. Plants flower in response to gibberellins, and DELLA proteins negatively regulate this gibberellin signaling (Fig. 4)^18^. Degradation of DELLAs is mediated by gibberellins. Out of the 20 genes, we were able to retrieve four genes, where pure epialleles possibly affected gene expression levels (Fig. 3). These four genes, including Vradi01g09470, Vradi01g11940, Vradi03g00420, and Vradi09g08580, showed the highest log_2_FC values (0.92, − 2.14, 1.86, and 2.28, respectively) between VC1973A and V2984, and their Arabidopsis orthologs have been well characterized. Vradi03g00420 encodes a gibberellin receptor, which is a positive regulator of the gibberellin-mediated signaling pathway in Arabidopsis (Table 3)^19^. At4G08850, an Arabidopsis ortholog of Vradi09g08580, which encodes a receptor-like protein, is regulated by DELLA proteins in flowering buds^18^. Vradi01g09470 and Vradi01g11940 are annotated as encoding a transmembrane protein and peroxisomal membrane protein, respectively, which may participate in communication among cells^20,21^. These results suggest that the expression levels of these genes are influenced by epigenetic factors and gibberellin-mediated signaling pathways involve in synchronicity of pod maturity in mungbean. About 1 kbp genomic regions around the DMRs for these four genes were confirmed by Sanger sequencing (Table S8).

In this study, we identified pure genic DMRs between high and low SPM groups in a mungbean RIL population. Although the data was collected without environmental repeat, phenotypic variation was normally distributed (*p*-value < 0.01), widely ranged from 0.319 to 0.862 among 187 lines. Based on pure epialleles independent of genetic variation, epigenetic factors underlying SPM in mungbean were identified, which would not be detected by nucleotide-based analysis. The data consistently indicated that various transcription factors and receptor proteins regulate SPM in mungbean via plant hormone (especially gibberellin)-mediated signaling pathways. However, our study has a limitation for estimating environmental variance of SPM, as phenotypic data was collected in a single environment without replication. Functional validation for the candidate genes responsible for SPM and phenotypic data with environmental repeats are still needed to fully elucidate SPM in mungbean. Overall, our results will help to understand the potential of epialleles as a possible breeding resource for improving the SPM phenotype of mungbean elite cultivars.

## Methods

### Plant materials and phenotyping

187 F_10_ RIL population derived from a cross between VC1973A (maternal parent) and V2984 (paternal parent) was planted at Seoul National University Experimental Farm in Suwon, Korea (37°16′12.7″N, 126°59′19.2″E) in 15 July 2016^22^. Natural photoperiod and average temperature from July to September 2016 in Suwon were 11.9–14.7 h per day and 22.7–27.7 °C, respectively. The mungbean cultivar VC1973A was developed at the World Vegetable Center (AVRDC) in 1982, and the Korean landrace V2984 is mostly grown in the Kyung-Ki province of South Korea. Genomic DNA was extracted from the first trifoliate leaf of ten mungbean genotypes, including eight RILs and two parental genotypes, using GeneAll Exgene Plant SV Kit (GeneAll Biotechnology, Co., Ltd., Korea). 10 plants for each genotype were harvested once a week for 8 weeks from August 26 to October 14, 2016^3^. The SPM index was calculated as the highest sum of pod numbers of 2 consecutive weeks divided by total pod numbers. Genotypes with the highest and lowest SPM indices were selected for bisulfite-sequencing and resequencing analyses. Resequencing was performed on the Illumina HiSeq4000 platform.

### Bisulfite-sequencing

Libraries for bisulfite-sequencing were constructed using TruSeq DNA Methylation Kit (Illumina, USA), after bisulfite conversion using Zymo EZ DNA Methylation Gold Kit (Zymo Research, USA), according to the manufacturer’s guidelines. Methyl-Seq was performed on the HiSeq X platform (Table S2). For each library construction, 0.1% lambda DNA (Invitrogen, USA) was added to plant genomic DNA. Methyl-Seq reads were mapped onto the in silico bisulfite-converted lambda DNA genome, and the efficiency of bisulfite conversion was calculated as the ratio of the number of converted cytosine residues to the total number of cytosine residues (including converted and unconverted).

### Identification of DMRs

Raw Methyl-Seq reads were mapped onto the in silico bisulfite-converted mungbean reference genome using Bismark-0.20.0^23^. To minimize potential clonal bias by PCR amplification, reads that mapped to the same position were removed. Cytosine residues with three or more reads were selected. DMRs were identified using Metilene with default parameters^24^. DMRs located within ± 2 kb of gene sequences were defined as genic DMRs. *Arabidopsis thaliana* orthologs of mungbean genes containing DMRs were mapped to the Kyoto Encyclopedia of Genes and Genomes database (KEGG; https://www.genome.jp/kegg/tool/map_pathway1.html)^13^ . GO enrichment was analyzed using BiNGO plugin in Cytoscape software^25^.

To calculate the methylation levels of genic DMRs, upstream (− 2 kb from the transcription start site), gene body, and downstream (+ 2 kb from the transcription termination site) sequences containing DMRs were divided into 20 bins. Average methylation levels of cytosine residues in three consecutive bins were calculated and plotted using a sliding window approach.

### Identification of pure DMRs

Illumina short reads generated using the Hiseq4000 platform were mapped against the mungbean reference genome (VC1973A) using Burrows-Wheeler Aligner (BWA) (Table S5)^5,26^. Single nucleotide polymorphisms (SNPs) and insertion/deletion mutations (Indels) were identified using Samtools with the following criteria: mapping quality > 30, minimum mapping depth ≥ 3, and maximum mapping depth ≤ 20 for RILs and ≤ 100 for V2984^27^. After the removal of duplicate variants, SNPs shared by all ten lines were used to identify pure DMRs. The association between methylation level and SNPs located within ± 1 kb of the DMRs was tested using Student’s *t*-test with *p*-value < 0.01. SNPs were annotated using SnpEff^28^. The DMRs that showed no significant association with SNPs were defined as pure DMRs.

## Supplementary information

Supplementary Information

## Data Availability

Resequencing and bisulfite sequencing data have been deposited at NCBI SRA database. All authors reviewed the manuscript.
