# Supplementary material for: Identification of epigenetic variation associated with synchronous pod maturity in mungbean (Vigna radiata L.)
Source: Sci Rep. 2020 Oct 15;10:17414. doi: 10.1038/s41598-020-74520-z (PMC7562708; doi:10.1038/s41598-020-74520-z)
Supplement: Supplementary file 1 — Supplementary Information [file 41598_2020_74520_MOESM1_ESM.pdf]

Identification of epigenetic variation associated with synchronous pod maturity in mungbean (*Vigna radiata* L.)

Jungmin Ha[1,2], Hakyung Kwon[1], Kang-Heum Cho[1], Min Young Yoon[1], Moon Young Kim[1,3], Suk-Ha Lee[1,3*]

[1]Department of Plant Science and Research Institute of Agriculture and Life Sciences, Seoul National University, Seoul 08826, Republic of Korea
[2]Department of Plant Science, Gangneung-Wonju National University, Gangneung, Republic of Korea
[3] Plant Genomics and Breeding Institute, Seoul National University, Seoul 08826, Republic of Korea

[*]Correspondence: Suk-Ha Lee
E-mail address: sukhalee@snu.ac.kr
Address: Department of Plant Science and Research Institute of Agriculture and Life Sciences, Seoul National University, Seoul 08826, Republic of Korea
Tel: +8228804545
Fax: +8228774550

Table S1. Phenotypic information for SPM index.

Parental lines[3] and eight lines with the highest and the lowest SPM index were indicated in bold.

	The number of pods weekly harvested											
	week1	week2	week3	week4	week5	week6	week7	week8	Total pod numbers	Days to first flower	Days to first pod maturity	SPM index
VC1973A	79	109	7	3	0	1	8	11	218	32	19	0.862
SK 10	102	164	14	4	2	13	25	12	336	33	20	0.792
SK 68	35	201	130	2	4	5	42	4	423	36	20	0.783
SK 186	140	80	27	4	1	8	15	10	285	34	18	0.772
SK 49	140	167	27	0	2	5	52	9	402	35	17	0.764
V2984	63	173	102	12	20	85	105	5	565	37	16	0.487
SK 94	40	126	27	8	36	78	91	15	421	34	18	0.394
SK 152	54	94	58	6	4	39	117	32	404	36	18	0.374
SK 176	113	95	123	5	12	84	134	33	599	40	25	0.364
SK 156	49	118	46	10	44	129	112	15	523	36	19	0.319

Table S2. Statistics for methy-sequencing raw reads.

0.1% lambda DNA was included to calculate conversion rate.

Genotype	Total Reads	Aligned Reads	Unique Reads	Conversion rate	NCBI SRA*
VC1973A	89,179,142	36,449,078	28,221,695	99.0	SAMN10915343
V2984	79,050,730	32,629,135	24,195,125	99.5	SAMN10915344
SK10	75,027,008	32,961,984	24,256,517	98.5	SAMN10915345
SK49	90,521,957	58,190,411	31,443,239	98.8	SAMN10915346
SK68	91,455,758	59,390,410	32,646,306	98.9	SAMN10915347
SK94	86,443,797	53,422,833	30,017,719	98.8	SAMN10915348
SK152	87,979,470	56,325,308	33,580,551	98.8	SAMN10915349
SK156	81,170,706	30,616,135	22,780,853	98.6	SAMN10915350
SK176	98,853,485	60,635,219	34,499,332	98.7	SAMN10915351
SK186	84,491,164	36,521,038	26,841,181	98.6	SAMN10915352

NCBI SRA*: National Center for Biotechnology Information Short Read Archive.

Table S3. KEGG pathway mapping of 211 genes proximal to genic DMRs.

Pathways	The number of genes
Metabolic pathways	20
Biosynthesis of secondary metabolites	7
Plant hormone signal transduction	4
Biosynthesis of amino acids	3
Peroxisome	3
Fatty acid biosynthesis	3
Endocytosis	3
Ubiquitin mediated proteolysis	3
Carbon metabolism	2
Amino sugar and nucleotide sugar metabolism	2
Aminoacyl-tRNA biosynthesis	2
Plant-pathogen interaction	2
Fatty acid metabolism	2
Glycine, serine and threonine metabolism	2
Oxidative phosphorylation	2
MAPK signaling pathway	2
Biotin metabolism	2
Phosphatidylinositol signaling system	2
Inositol phosphate metabolism	2
Protein processing in endoplasmic reticulum	2
Phagosome	2
DNA replication	2
Purine metabolism	2

Table S4. Average methylation levels of genic DMRs.

hhDMRs consist of the genic DMRs where methylation level is significantly higher in high SPM group than low SPM group. hlDMRs consist of the genic DMRs where methylation level is significantly higher in low SPM group than high SPM group.

	Methylation type	High synchronous group (%)	Low synchronous group (%)	Differences (%)
hhDMRs	CpG	64.1	49.5	14.7
	CHG	50.0	43.4	6.6
	CHH	11.7	10.5	1.1
hlDMRs	CpG	39.4	55.7	16.3
	CHG	31.3	41.3	9.9
	CHH	9.3	12.0	2.7

Table S5. Statistics for re-sequencing raw reads.

Nucleotide variations were filtered by Samtools (mapping quality > 30, minimum mapping depth ≥ 3, maximum mapping depth ≤ 20 for RILs and 100 for V2984).

SPM	Genotype	Total bases	Average mapping depth	Coverage	The number of SNPs	The number of INDELs	NCBI SRA
High	VC1973A	Reference (Kang et al., 2014)					
	SK10	5,560,612,245	10.7	90%	372,293	42,377	SAMN10915353
	SK68	5,592,400,393	10.9	90%	359,206	41,873	SAMN10915354
	SK186	5,053,846,784	9.0	90%	281,020	29,714	SAMN10915355
	SK49	3,891,876,381	7.1	89%	268,604	25,908	SAMN10915356
Low	V2984*	34,961,160,635	82.3	91%	797,865	134,082	Kang et al., 2014
	SK94	5,360,257,036	9.7	89%	449,293	48,209	SAMN10915357
	SK152	5,362,006,578	9.2	89%	524,113	57,556	SAMN10915358
	SK176	5,316,372,168	9.1	89%	549,844	57,457	SAMN10915359
	SK156	5,471,133,798	9.5	90%	397,837	43,735	SAMN10915360

V2984*: Illumina raw reads are available at NCBI SRA (Kang et al., 2014)

Table S6. The list of 544 SNPs within 2kbp of upstream and downstream of the genes proximal to DMRs.

*Impact: Impact of SNPs determined by SnpEff.

Gene	Location	Position	*Impact	Gene	Location	Position	*Impact	Gene	Location	Position	*Impact
Vradi01g09470	16949066	downstream	MODIFIER	Vradi02g11080	20763540	upstream	MODIFIER	Vradi07g01900	3196239	intron	MODIFIER
Vradi01g09470	16949168	downstream	MODIFIER	Vradi03g00420	699233	intron	MODIFIER	Vradi07g01900	3196243	intron	MODIFIER
Vradi01g09470	16949176	downstream	MODIFIER	Vradi03g00420	699367	intron	MODIFIER	Vradi07g01900	3196414	intron	MODIFIER
Vradi01g09470	16949312	downstream	MODIFIER	Vradi03g00420	701292	downstream	MODIFIER	Vradi07g01900	3196544	intron	MODIFIER
Vradi01g09470	16949323	downstream	MODIFIER	Vradi04g01340	3930535	upstream	MODIFIER	Vradi07g01900	3196953	intron	MODIFIER
Vradi01g09470	16949365	downstream	MODIFIER	Vradi04g01340	3930635	upstream	MODIFIER	Vradi07g01900	3196970	intron	MODIFIER
Vradi01g09470	16949375	downstream	MODIFIER	Vradi04g01340	3930644	upstream	MODIFIER	Vradi07g01900	3196980	intron	MODIFIER
Vradi01g09470	16949440	downstream	MODIFIER	Vradi04g01340	3930716	upstream	MODIFIER	Vradi07g01900	3197963	intron	MODIFIER
Vradi01g09470	16949475	downstream	MODIFIER	Vradi06g07570	11116346	intron	MODIFIER	Vradi07g01900	3197981	intron	MODIFIER
Vradi01g09470	16949479	downstream	MODIFIER	Vradi06g07570	11121271	intron	MODIFIER	Vradi07g01900	3197982	intron	MODIFIER
Vradi01g09470	16949488	downstream	MODIFIER	Vradi06g07570	11125833	intron	MODIFIER	Vradi07g01900	3198018	intron	MODIFIER
Vradi01g09470	16949727	downstream	MODIFIER	Vradi06g07580	11125833	upstream	MODIFIER	Vradi07g01900	3198187	intron	MODIFIER
Vradi01g09470	16949729	downstream	MODIFIER	Vradi06g09130	18541779	upstream	MODIFIER	Vradi07g01900	3198242	intron	MODIFIER
Vradi01g09470	16949829	downstream	MODIFIER	Vradi06g09130	18541834	upstream	MODIFIER	Vradi07g01900	3198280	intron	MODIFIER
Vradi01g09470	16949839	downstream	MODIFIER	Vradi06g09130	18541905	upstream	MODIFIER	Vradi07g01900	3198323	intron	MODIFIER
Vradi01g09470	16949913	downstream	MODIFIER	Vradi06g09130	18542011	upstream	MODIFIER	Vradi07g01900	3198327	intron	MODIFIER
Vradi01g09470	16950040	downstream	MODIFIER	Vradi06g09130	18542132	upstream	MODIFIER	Vradi07g01900	3200934	intron	MODIFIER
Vradi01g09470	16950217	downstream	MODIFIER	Vradi06g09130	18542193	upstream	MODIFIER	Vradi07g01900	3201043	intron	MODIFIER
Vradi01g09470	16950921	downstream	MODIFIER	Vradi06g09130	18542250	upstream	MODIFIER	Vradi07g01900	3201697	intron	MODIFIER
Vradi01g09470	16951412	intron	MODIFIER	Vradi06g09130	18542343	missense	MODERATE	Vradi07g01900	3202068	intron	MODIFIER
Vradi01g09470	16951428	intron	MODIFIER	Vradi06g09130	18542728	synonymous	LOW	Vradi07g01900	3202087	intron	MODIFIER
Vradi01g09470	16951512	intron	MODIFIER	Vradi06g09130	18542884	synonymous	LOW	Vradi07g01900	3202151	intron	MODIFIER
Vradi01g09470	16951535	intron	MODIFIER	Vradi06g09130	18542911	synonymous	LOW	Vradi07g01900	3202172	intron	MODIFIER
Vradi01g09470	16952902	synonymous	LOW	Vradi06g09130	18543650	downstream	MODIFIER	Vradi07g01900	3202649	intron	MODIFIER
Vradi01g09470	16953489	intron	MODIFIER	Vradi06g09130	18544110	downstream	MODIFIER	Vradi07g01900	3202835	intron	MODIFIER
Vradi01g09470	16954690	upstream	MODIFIER	Vradi06g09130	18544203	downstream	MODIFIER	Vradi07g01900	3202895	intron	MODIFIER
Vradi01g09470	16954765	upstream	MODIFIER	Vradi06g11970	28848336	downstream	MODIFIER	Vradi07g01900	3205344	intron	MODIFIER

Vradi01g09470	16954826	upstream	MODIFIER	Vradi06g11970	28851602	missense	MODERATE	Vradi07g01900	3205592	intron	MODIFIER
Vradi01g09470	16955541	upstream	MODIFIER	Vradi06g11970	28854542	upstream	MODIFIER	Vradi07g01900	3206322	intron	MODIFIER
Vradi01g09470	16955586	upstream	MODIFIER	Vradi06g11970	28855479	upstream	MODIFIER	Vradi07g01900	3207543	intron	MODIFIER
Vradi01g09470	16955766	upstream	MODIFIER	Vradi06g11970	28856076	upstream	MODIFIER	Vradi07g01900	3208093	intron	MODIFIER
Vradi01g09470	16955895	upstream	MODIFIER	Vradi06g11970	28856312	upstream	MODIFIER	Vradi07g01900	3208218	intron	MODIFIER
Vradi01g09470	16956205	upstream	MODIFIER	Vradi06g12210	29573910	upstream	MODIFIER	Vradi07g01900	3208490	intron	MODIFIER
Vradi01g09470	16956229	upstream	MODIFIER	Vradi06g12210	29573927	upstream	MODIFIER	Vradi07g01900	3209159	intron	MODIFIER
Vradi01g09470	16956279	upstream	MODIFIER	Vradi06g12210	29573949	upstream	MODIFIER	Vradi07g01900	3209214	intron	MODIFIER
Vradi01g09470	16956280	upstream	MODIFIER	Vradi06g12210	29573958	upstream	MODIFIER	Vradi07g01900	3209367	intron	MODIFIER
Vradi01g09470	16956283	upstream	MODIFIER	Vradi06g12210	29573985	upstream	MODIFIER	Vradi07g01900	3209400	intron	MODIFIER
Vradi01g09470	16956297	upstream	MODIFIER	Vradi06g12210	29574236	upstream	MODIFIER	Vradi07g01900	3209676	intron	MODIFIER
Vradi01g09470	16956302	upstream	MODIFIER	Vradi06g12210	29574240	upstream	MODIFIER	Vradi07g01900	3209864	intron	MODIFIER
Vradi01g09470	16956308	upstream	MODIFIER	Vradi06g12210	29574246	upstream	MODIFIER	Vradi07g01900	3210069	intron	MODIFIER
Vradi01g09470	16956309	upstream	MODIFIER	Vradi06g12210	29574750	upstream	MODIFIER	Vradi07g01900	3210093	intron	MODIFIER
Vradi01g09470	16956316	upstream	MODIFIER	Vradi06g12210	29574886	upstream	MODIFIER	Vradi07g01900	3210116	intron	MODIFIER
Vradi01g09510	17042606	missense	MODERATE	Vradi06g12210	29576793	intron	MODIFIER	Vradi07g01900	3210133	intron	MODIFIER
Vradi01g09510	17043382	intron	MODIFIER	Vradi06g12210	29576983	intron	MODIFIER	Vradi07g01900	3210978	upstream	MODIFIER
Vradi01g09510	17044126	intron	MODIFIER	Vradi06g12210	29577005	intron	MODIFIER	Vradi07g01900	3211044	upstream	MODIFIER
Vradi01g09510	17044359	intron	MODIFIER	Vradi06g12210	29577296	intron	MODIFIER	Vradi07g01900	3211087	upstream	MODIFIER
Vradi01g09510	17044505	intron	MODIFIER	Vradi06g12210	29577641	missense	MODERATE	Vradi07g01900	3211643	upstream	MODIFIER
Vradi01g09510	17045627	intron	MODIFIER	Vradi06g12210	29578572	intron	MODIFIER	Vradi07g01900	3211678	upstream	MODIFIER
Vradi01g09510	17045671	intron	MODIFIER	Vradi06g12210	29578700	intron	MODIFIER	Vradi07g01900	3211680	upstream	MODIFIER
Vradi01g09510	17045676	intron	MODIFIER	Vradi06g12210	29581213	intron	MODIFIER	Vradi07g01900	3211685	upstream	MODIFIER
Vradi01g09510	17045700	intron	MODIFIER	Vradi06g12210	29581305	intron	MODIFIER	Vradi07g01900	3211708	upstream	MODIFIER
Vradi01g09510	17045832	intron	MODIFIER	Vradi06g12210	29581653	intron	MODIFIER	Vradi07g01900	3211749	upstream	MODIFIER
Vradi01g09510	17045849	intron	MODIFIER	Vradi06g12210	29582177	intron	MODIFIER	Vradi07g01900	3211957	upstream	MODIFIER
Vradi01g09510	17046252	intron	MODIFIER	Vradi06g12210	29582179	intron	MODIFIER	Vradi07g01900	3211969	upstream	MODIFIER
Vradi01g09510	17046498	intron	MODIFIER	Vradi06g12210	29582665	intron	MODIFIER	Vradi07g01910	3209159	upstream	MODIFIER
Vradi01g09510	17046541	intron	MODIFIER	Vradi06g12210	29582698	intron	MODIFIER	Vradi07g01910	3209214	upstream	MODIFIER
Vradi01g09510	17046605	intron	MODIFIER	Vradi06g12210	29582906	intron	MODIFIER	Vradi07g01910	3209367	upstream	MODIFIER
Vradi01g09510	17046641	intron	MODIFIER	Vradi06g12210	29583258	intron	MODIFIER	Vradi07g01910	3209400	upstream	MODIFIER

Vradi01g09510	17047825	intron	MODIFIER	Vradi06g12210	29583597	intron	MODIFIER	Vradi07g01910	3209676	upstream	MODIFIER
Vradi01g09510	17048486	intron	MODIFIER	Vradi06g12210	29583804	intron	MODIFIER	Vradi07g01910	3209864	upstream	MODIFIER
Vradi01g09510	17049328	intron	MODIFIER	Vradi06g12210	29584116	intron	MODIFIER	Vradi07g01910	3210069	upstream	MODIFIER
Vradi01g09510	17050752	intron	MODIFIER	Vradi06g12210	29584529	intron	MODIFIER	Vradi07g01910	3210093	upstream	MODIFIER
Vradi01g09510	17050800	intron	MODIFIER	Vradi06g12210	29584834	intron	MODIFIER	Vradi07g01910	3210116	upstream	MODIFIER
Vradi01g09510	17050974	intron	MODIFIER	Vradi06g12210	29584839	intron	MODIFIER	Vradi07g01910	3210133	upstream	MODIFIER
Vradi01g09510	17052528	intron	MODIFIER	Vradi06g12210	29584845	intron	MODIFIER	Vradi07g01910	3210978	upstream	MODIFIER
Vradi01g09510	17052757	synonymous	LOW	Vradi06g12210	29585488	intron	MODIFIER	Vradi07g01910	3211044	upstream	MODIFIER
Vradi01g09510	17053342	intron	MODIFIER	Vradi06g12210	29585506	intron	MODIFIER	Vradi07g01910	3211087	upstream	MODIFIER
Vradi01g09510	17054698	downstream	MODIFIER	Vradi06g12210	29585551	intron	MODIFIER	Vradi07g01910	3211643	upstream	MODIFIER
Vradi01g09510	17054804	downstream	MODIFIER	Vradi06g12210	29585729	intron	MODIFIER	Vradi07g01910	3211678	upstream	MODIFIER
Vradi01g09510	17055092	downstream	MODIFIER	Vradi06g12210	29585958	intron	MODIFIER	Vradi07g01910	3211680	upstream	MODIFIER
Vradi01g09510	17055098	downstream	MODIFIER	Vradi06g12210	29585959	intron	MODIFIER	Vradi07g01910	3211685	upstream	MODIFIER
Vradi01g09510	17055147	downstream	MODIFIER	Vradi06g12210	29585961	intron	MODIFIER	Vradi07g01910	3211708	upstream	MODIFIER
Vradi01g09510	17055202	downstream	MODIFIER	Vradi06g12210	29586124	intron	MODIFIER	Vradi07g01910	3211749	upstream	MODIFIER
Vradi01g09510	17055386	downstream	MODIFIER	Vradi06g12210	29586625	intron	MODIFIER	Vradi07g01910	3211957	upstream	MODIFIER
Vradi01g09510	17055423	downstream	MODIFIER	Vradi06g12210	29586691	intron	MODIFIER	Vradi07g01910	3211969	upstream	MODIFIER
Vradi01g09510	17055437	downstream	MODIFIER	Vradi06g12210	29588059	intron	MODIFIER	Vradi07g23770	46916379	upstream	MODIFIER
Vradi01g09510	17055452	downstream	MODIFIER	Vradi06g12210	29588616	intron	MODIFIER	Vradi07g23770	46917336	upstream	MODIFIER
Vradi01g09510	17055569	downstream	MODIFIER	Vradi06g12210	29588623	intron	MODIFIER	Vradi08g04830	9300069	intron	MODIFIER
Vradi01g09510	17055578	downstream	MODIFIER	Vradi06g12210	29588893	intron	MODIFIER	Vradi08g04830	9305240	upstream	MODIFIER
Vradi01g09510	17055584	downstream	MODIFIER	Vradi06g12210	29588991	intron	MODIFIER	Vradi08g04830	9305808	upstream	MODIFIER
Vradi01g09510	17055819	downstream	MODIFIER	Vradi06g12210	29589005	intron	MODIFIER	Vradi08g04840	9300069	upstream	MODIFIER
Vradi01g09510	17055841	downstream	MODIFIER	Vradi06g12210	29589209	missense	MODERATE	Vradi08g04840	9305240	synonymous	LOW
Vradi01g09510	17055845	downstream	MODIFIER	Vradi06g12210	29589354	missense	MODERATE	Vradi08g04840	9305808	synonymous	LOW
Vradi01g09510	17055885	downstream	MODIFIER	Vradi06g12210	29589382	synonymous	LOW	Vradi08g04840	9307396	downstream	MODIFIER
Vradi01g09510	17056006	downstream	MODIFIER	Vradi06g12210	29589507	missense	MODERATE	Vradi08g04840	9307399	downstream	MODIFIER
Vradi01g09510	17056055	downstream	MODIFIER	Vradi06g12210	29589850	missense	MODERATE	Vradi08g18940	40788651	upstream	MODIFIER
Vradi01g09510	17056090	downstream	MODIFIER	Vradi06g12210	29590134	missense	MODERATE	Vradi08g18940	40788944	upstream	MODIFIER
Vradi01g09510	17056199	downstream	MODIFIER	Vradi06g12210	29590159	missense	MODERATE	Vradi08g18940	40789610	intron	MODIFIER
Vradi01g09510	17056219	downstream	MODIFIER	Vradi06g12210	29590189	missense	MODERATE	Vradi08g18940	40789809	intron	MODIFIER

Vradi01g09510	17056296	downstream	MODIFIER	Vradi06g12210	29590223	synonymous	LOW	Vradi08g18940	40789834	intron	MODIFIER
Vradi01g11940	24419879	upstream	MODIFIER	Vradi06g12210	29590346	intron	MODIFIER	Vradi08g18940	40791049	intron	MODIFIER
Vradi01g11940	24420029	upstream	MODIFIER	Vradi06g12210	29590388	missense & splice region	MODERATE	Vradi08g18940	40791686	intron	MODIFIER
Vradi01g11940	24420078	upstream	MODIFIER	Vradi06g12210	29590389	synonymous	LOW	Vradi08g18940	40792064	synonymous	LOW
Vradi01g11940	24420917	upstream	MODIFIER	Vradi06g12210	29591414	intron	MODIFIER	Vradi08g18940	40793834	downstream	MODIFIER
Vradi01g11940	24420931	upstream	MODIFIER	Vradi06g12210	29591716	intron	MODIFIER	Vradi08g18940	40793835	downstream	MODIFIER
Vradi01g11940	24421151	upstream	MODIFIER	Vradi06g12210	29591719	intron	MODIFIER	Vradi08g18940	40793863	downstream	MODIFIER
Vradi01g11940	24421245	missense & splice region	MODERATE	Vradi06g12210	29591760	intron	MODIFIER	Vradi08g18940	40793864	downstream	MODIFIER
Vradi01g11940	24421658	missense	MODERATE	Vradi06g12210	29591953	intron	MODIFIER	Vradi08g18940	40794046	downstream	MODIFIER
Vradi01g11940	24421988	intron	MODIFIER	Vradi06g12210	29591991	intron	MODIFIER	Vradi08g18940	40794058	downstream	MODIFIER
Vradi01g11940	24422025	intron	MODIFIER	Vradi06g12210	29592028	intron	MODIFIER	Vradi08g18940	40794865	downstream	MODIFIER
Vradi01g11940	24422027	intron	MODIFIER	Vradi06g12210	29592258	intron	MODIFIER	Vradi08g18940	40794983	downstream	MODIFIER
Vradi01g11940	24422029	intron	MODIFIER	Vradi06g12210	29592277	intron	MODIFIER	Vradi08g18940	40794990	downstream	MODIFIER
Vradi01g11940	24422033	intron	MODIFIER	Vradi06g12210	29592334	intron	MODIFIER	Vradi08g18940	40795126	downstream	MODIFIER
Vradi01g11940	24422046	intron	MODIFIER	Vradi06g12210	29592377	intron	MODIFIER	Vradi08g18940	40795146	downstream	MODIFIER
Vradi01g11940	24422047	intron	MODIFIER	Vradi06g12210	29592382	intron	MODIFIER	Vradi09g08580	15658686	upstream	MODIFIER
Vradi01g11940	24422643	intron	MODIFIER	Vradi06g12210	29593008	intron	MODIFIER	Vradi09g08580	15658783	upstream	MODIFIER
Vradi01g11940	24422882	intron	MODIFIER	Vradi06g12210	29593064	intron	MODIFIER	Vradi09g08580	15658785	upstream	MODIFIER
Vradi01g11940	24423145	intron	MODIFIER	Vradi06g12210	29593182	intron	MODIFIER	Vradi09g08580	15658863	upstream	MODIFIER
Vradi01g11940	24423386	intron	MODIFIER	Vradi06g12210	29593183	intron	MODIFIER	Vradi09g08580	15658924	upstream	MODIFIER
Vradi01g11940	24423436	intron	MODIFIER	Vradi06g12210	29593250	intron	MODIFIER	Vradi09g08580	15659005	upstream	MODIFIER
Vradi01g11940	24423463	intron	MODIFIER	Vradi06g12210	29594761	missense	MODERATE	Vradi09g08580	15659031	upstream	MODIFIER
Vradi01g11940	24423480	intron	MODIFIER	Vradi06g12210	29595226	downstream	MODIFIER	Vradi09g08580	15659040	upstream	MODIFIER
Vradi01g11940	24424640	missense	MODERATE	Vradi06g12210	29595274	downstream	MODIFIER	Vradi09g08580	15659229	upstream	MODIFIER
Vradi01g11940	24424936	intron	MODIFIER	Vradi06g12210	29596189	downstream	MODIFIER	Vradi09g08580	15659248	upstream	MODIFIER
Vradi01g11940	24425182	intron	MODIFIER	Vradi06g12210	29596234	downstream	MODIFIER	Vradi09g08580	15659295	upstream	MODIFIER
Vradi01g11940	24425404	intron	MODIFIER	Vradi06g12210	29596430	downstream	MODIFIER	Vradi09g08580	15659301	upstream	MODIFIER
Vradi01g11940	24425449	intron	MODIFIER	Vradi06g12210	29596438	downstream	MODIFIER	Vradi09g08580	15659302	upstream	MODIFIER
Vradi01g11940	24425487	intron	MODIFIER	Vradi06g12210	29596491	downstream	MODIFIER	Vradi09g08580	15660221	upstream	MODIFIER
Vradi01g11940	24425495	intron	MODIFIER	Vradi07g01890	3187659	upstream	MODIFIER	Vradi09g08580	15660226	upstream	MODIFIER
Vradi01g11940	24425736	intron	MODIFIER	Vradi07g01890	3187872	upstream	MODIFIER	Vradi09g08580	15660494	upstream	MODIFIER

Vradi01g11940	24425844	intron	MODIFIER	Vradi07g01890	3188157	upstream	MODIFIER	Vradi09g08580	15660587	missense	MODERATE
Vradi01g11940	24425946	intron	MODIFIER	Vradi07g01890	3188166	upstream	MODIFIER	Vradi09g08580	15660686	missense	MODERATE
Vradi01g11940	24425979	intron	MODIFIER	Vradi07g01890	3188201	upstream	MODIFIER	Vradi09g08580	15660836	missense	MODERATE
Vradi01g11940	24426318	intron	MODIFIER	Vradi07g01890	3188219	upstream	MODIFIER	Vradi09g08580	15661104	synonymous	LOW
Vradi01g11940	24426560	intron	MODIFIER	Vradi07g01890	3188239	upstream	MODIFIER	Vradi09g08580	15661125	missense	MODERATE
Vradi01g11940	24427101	intron	MODIFIER	Vradi07g01890	3188246	upstream	MODIFIER	Vradi09g08580	15661140	synonymous	LOW
Vradi01g11940	24427162	intron	MODIFIER	Vradi07g01890	3188294	upstream	MODIFIER	Vradi09g08580	15661161	synonymous	LOW
Vradi01g11940	24427395	intron	MODIFIER	Vradi07g01890	3188349	upstream	MODIFIER	Vradi09g08580	15661225	intron	MODIFIER
Vradi01g11940	24427674	intron	MODIFIER	Vradi07g01890	3188550	upstream	MODIFIER	Vradi09g08580	15661261	missense	MODERATE
Vradi01g11940	24427690	intron	MODIFIER	Vradi07g01890	3188818	upstream	MODIFIER	Vradi09g08580	15661269	synonymous	LOW
Vradi01g11940	24427911	intron	MODIFIER	Vradi07g01890	3189022	upstream	MODIFIER	Vradi09g08580	15661320	synonymous	LOW
Vradi01g11940	24428010	intron	MODIFIER	Vradi07g01890	3189415	upstream	MODIFIER	Vradi09g08580	15661380	synonymous	LOW
Vradi01g11940	24428310	intron	MODIFIER	Vradi07g01890	3189725	upstream	MODIFIER	Vradi09g08580	15661420	missense	MODERATE
Vradi01g11940	24428374	intron	MODIFIER	Vradi07g01890	3190264	upstream	MODIFIER	Vradi09g08580	15661611	synonymous	LOW
Vradi01g11940	24428802	intron	MODIFIER	Vradi07g01890	3190921	upstream	MODIFIER	Vradi09g08580	15661648	missense	MODERATE
Vradi01g11940	24429180	intron	MODIFIER	Vradi07g01890	3191320	upstream	MODIFIER	Vradi09g08580	15661802	stop gained	HIGH
Vradi01g11940	24429264	intron	MODIFIER	Vradi07g01900	3187659	downstream	MODIFIER	Vradi09g08580	15661815	synonymous	LOW
Vradi01g11940	24429281	intron	MODIFIER	Vradi07g01900	3187872	downstream	MODIFIER	Vradi09g08580	15661905	synonymous	LOW
Vradi01g11940	24429284	intron	MODIFIER	Vradi07g01900	3188157	downstream	MODIFIER	Vradi09g08580	15661945	missense	MODERATE
Vradi01g11940	24429672	intron	MODIFIER	Vradi07g01900	3188166	downstream	MODIFIER	Vradi09g08580	15662114	missense	MODERATE
Vradi01g11940	24429744	intron	MODIFIER	Vradi07g01900	3188201	downstream	MODIFIER	Vradi09g08580	15662125	missense	MODERATE
Vradi01g11940	24429776	intron	MODIFIER	Vradi07g01900	3188219	downstream	MODIFIER	Vradi09g08580	15662354	downstream	MODIFIER
Vradi01g11940	24430320	intron	MODIFIER	Vradi07g01900	3188239	downstream	MODIFIER	Vradi09g08580	15662429	downstream	MODIFIER
Vradi01g11940	24430736	intron	MODIFIER	Vradi07g01900	3188246	downstream	MODIFIER	Vradi09g08580	15662623	downstream	MODIFIER
Vradi01g11940	24431551	intron	MODIFIER	Vradi07g01900	3188294	downstream	MODIFIER	Vradi09g08580	15662633	downstream	MODIFIER
Vradi01g11940	24433258	downstream	MODIFIER	Vradi07g01900	3188349	downstream	MODIFIER	Vradi09g08580	15662685	downstream	MODIFIER
Vradi01g11940	24433493	downstream	MODIFIER	Vradi07g01900	3188550	downstream	MODIFIER	Vradi09g08580	15662696	downstream	MODIFIER
Vradi01g11940	24433553	downstream	MODIFIER	Vradi07g01900	3188818	downstream	MODIFIER	Vradi09g08580	15663099	downstream	MODIFIER
Vradi01g11950	24433493	downstream	MODIFIER	Vradi07g01900	3189022	downstream	MODIFIER	Vradi09g08580	15663117	downstream	MODIFIER
Vradi01g11950	24433553	downstream	MODIFIER	Vradi07g01900	3189415	3’ UTR	MODIFIER	Vradi09g08580	15663283	downstream	MODIFIER
Vradi01g12060	24890612	upstream	MODIFIER	Vradi07g01900	3189725	synonymous	LOW	Vradi09g08580	15663914	downstream	MODIFIER

Vradi01g12060	24890687	upstream	MODIFIER	Vradi07g01900	3190264	intron	MODIFIER	Vradi09g08580	15664064	downstream	MODIFIER
Vradi01g12060	24890719	upstream	MODIFIER	Vradi07g01900	3190921	intron	MODIFIER	Vradi09g08580	15664087	downstream	MODIFIER
Vradi01g12060	24891098	upstream	MODIFIER	Vradi07g01900	3191320	intron	MODIFIER	Vradi09g08580	15664093	downstream	MODIFIER
Vradi01g12060	24891099	upstream	MODIFIER	Vradi07g01900	3191976	intron	MODIFIER	Vradi11g00660	667117	downstream	MODIFIER
Vradi01g12060	24891813	upstream	MODIFIER	Vradi07g01900	3192514	intron	MODIFIER	Vradi11g00660	667322	downstream	MODIFIER
Vradi01g12060	24892373	intron	MODIFIER	Vradi07g01900	3192608	intron	MODIFIER	Vradi11g00660	667333	downstream	MODIFIER
Vradi01g12060	24894358	intron	MODIFIER	Vradi07g01900	3193535	intron	MODIFIER	Vradi11g00660	667395	downstream	MODIFIER
Vradi01g12060	24894381	intron	MODIFIER	Vradi07g01900	3193544	intron	MODIFIER	Vradi11g00660	667454	downstream	MODIFIER
Vradi01g12060	24894583	missense	MODERATE	Vradi07g01900	3193574	intron	MODIFIER	Vradi11g00660	667460	downstream	MODIFIER
Vradi01g12060	24894619	missense	MODERATE	Vradi07g01900	3193581	intron	MODIFIER	Vradi11g00660	667468	downstream	MODIFIER
Vradi01g12060	24894772	synonymous	LOW	Vradi07g01900	3193588	intron	MODIFIER	Vradi11g00670	667117	downstream	MODIFIER
Vradi01g12060	24895615	downstream	MODIFIER	Vradi07g01900	3193780	intron	MODIFIER	Vradi11g00670	667322	downstream	MODIFIER
Vradi01g12060	24895860	downstream	MODIFIER	Vradi07g01900	3193982	intron	MODIFIER	Vradi11g00670	667333	downstream	MODIFIER
Vradi01g12060	24895926	downstream	MODIFIER	Vradi07g01900	3194021	intron	MODIFIER	Vradi11g00670	667395	downstream	MODIFIER
Vradi01g12060	24896229	downstream	MODIFIER	Vradi07g01900	3194029	intron	MODIFIER	Vradi11g00670	667454	downstream	MODIFIER
Vradi01g12060	24896252	downstream	MODIFIER	Vradi07g01900	3194224	intron	MODIFIER	Vradi11g00670	667460	downstream	MODIFIER
Vradi01g12060	24896370	downstream	MODIFIER	Vradi07g01900	3194280	intron	MODIFIER	Vradi11g00670	667468	downstream	MODIFIER
Vradi01g12060	24897176	downstream	MODIFIER	Vradi07g01900	3194498	intron	MODIFIER	Vradi11g00670	672003	intron	MODIFIER
Vradi01g12060	24897273	downstream	MODIFIER	Vradi07g01900	3194585	intron	MODIFIER	Vradi11g00670	672069	intron	MODIFIER
Vradi02g11070	20754279	downstream	MODIFIER	Vradi07g01900	3194633	intron	MODIFIER	Vradi11g00670	672123	intron	MODIFIER
Vradi02g11070	20755042	downstream	MODIFIER	Vradi07g01900	3194648	intron	MODIFIER	Vradi11g00670	672406	intron	MODIFIER
Vradi02g11070	20755817	downstream	MODIFIER	Vradi07g01900	3194699	intron	MODIFIER	Vradi11g00670	672509	intron	MODIFIER
Vradi02g11080	20754279	intron	MODIFIER	Vradi07g01900	3195157	intron	MODIFIER	Vradi11g00670	672916	intron	MODIFIER
Vradi02g11080	20755042	intron	MODIFIER	Vradi07g01900	3195160	intron	MODIFIER	Vradi11g00670	672918	intron	MODIFIER
Vradi02g11080	20755817	intron	MODIFIER	Vradi07g01900	3195252	intron	MODIFIER	Vradi11g00670	672945	intron	MODIFIER
Vradi02g11080	20758088	intron	MODIFIER	Vradi07g01900	3196100	intron	MODIFIER	Vradi11g00670	672953	intron	MODIFIER
Vradi02g11080	20758211	intron	MODIFIER	Vradi07g01900	3196129	intron	MODIFIER	Vradi11g00670	672972	intron	MODIFIER
Vradi02g11080	20758217	intron	MODIFIER	Vradi07g01900	3196137	intron	MODIFIER	Vradi11g00670	673457	upstream	MODIFIER
Vradi02g11080	20759115	intron	MODIFIER	Vradi07g01900	3196138	intron	MODIFIER	Vradi11g00680	673457	downstream	MODIFIER
Vradi02g11080	20760746	intron	MODIFIER	Vradi07g01900	3196155	intron	MODIFIER				
Vradi02g11080	20763370	upstream	MODIFIER	Vradi07g01900	3196216	intron	MODIFIER				

Table S7. List of transcription factors proximal to DMRs.

Gene name	*At* orthologs	Domain	Description
Vradi0284s00060	AT2G33860	ARF	auxin response factor, putative, expressed
Vradi0347s00090	AT1G59640	bHLH	BEE 1, putative, expressed
Vradi07g03990	AT1G45249	bZIP	bZIP transcription factor, putative, expressed
Vradi08g06860	AT3G23240	ERF	AP2 domain containing protein, expressed
Vradi0332s00010	AT3G07500	FAR1	FAR1 family protein, expressed
Vradi09g08610	AT4G38180	FAR1	transposon protein, putative, unclassified, expressed
Vradi0171s00090	AT4G00150	GRAS	scarecrow transcription factor family protein, putative, expressed
Vradi02g10380	AT3G18380	HB-other	retrotransposon protein, putative, unclassified, expressed
Vradi0227s00040	AT1G31320	LBD	DUF260 domain containing protein, putative, expressed
Vradi07g10830	AT4G37260	MYB	MYB family transcription factor, putative, expressed
Vradi06g11270	AT1G01720	NAC	No apical meristem protein, putative, expressed
Vradi07g16030	AT1G61110	NAC	no apical meristem protein, putative, expressed
Vradi0341s00020	AT1G69490	NAC	No apical meristem protein, putative, expressed
Vradi03g10220	AT5G05660	NF-X1	NF-X1-type zinc finger protein, putative, expressed
Vradi01g14220	AT4G24020	Nin-like	NIN, putative, expressed
Vradi02g12120	AT1G13450	Trihelix	MYB family transcription factor, putative, expressed
Vradi01g11520	AT1G62300	WRKY	WRKY1, expressed

Table S8. List of primer sequences for Sanger sequencing.

primer ID	sequence	target DMRs	product size (bp)
Vradi01g09470_F	GGCCATTGACCCCATTAAAGTT	Vr01:6955933-16956106	1308
Vradi01g09470_R	CTAATGGTGCTCACAGAGCCA		
Vradi01g11940_F	GGTTGTTTGCGCCACAAGTAT	Vr01:24430241-24430310	1152
Vradi01g11940_R	TGTGGGTGCATGAGTCTACAAG		
Vradi03g00420_F	AAACGTTGGCTCGTTTTACCG	Vr03:698270-698471	1100
Vradi03g00420_R	GAACAGGAAGCAGTTGTCCG		
Vradi09g08580_F	TGCACCGAGTTACAGAGGAAAA	Vr09:15662981-15663298	1223
Vradi09g08580_R	TGTGCAACTGAGCATGGTGTAA	Vr09:15663317-15663764	

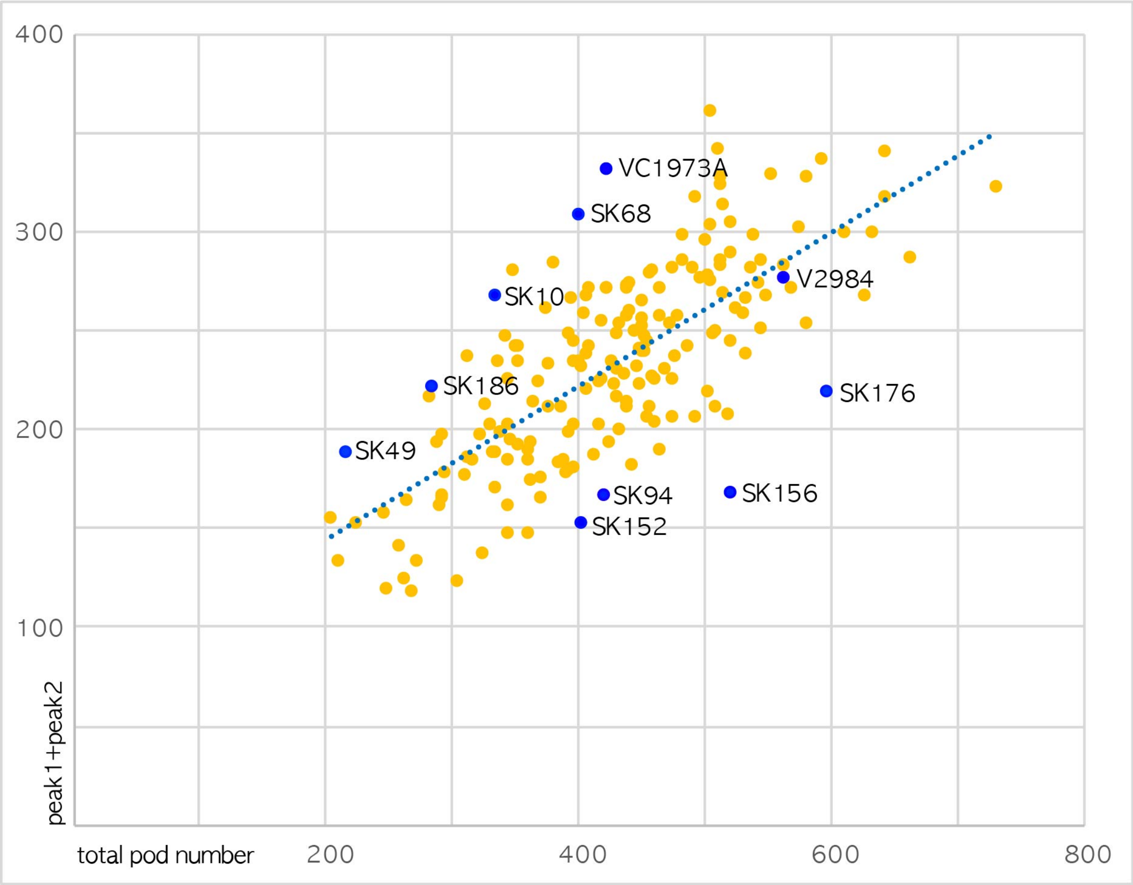

Figure S1. Distribution of SPM of 187 RIL lines. X and y axis indicate total pod numbers and the highest sum of pod numbers of two consecutive weeks, respectively. Trend line was represented as dotted line. Among 187 RILs, four lines with the highest SPM index and four lines with the lowest SPM index, including parental lines, were indicated with blue dots.

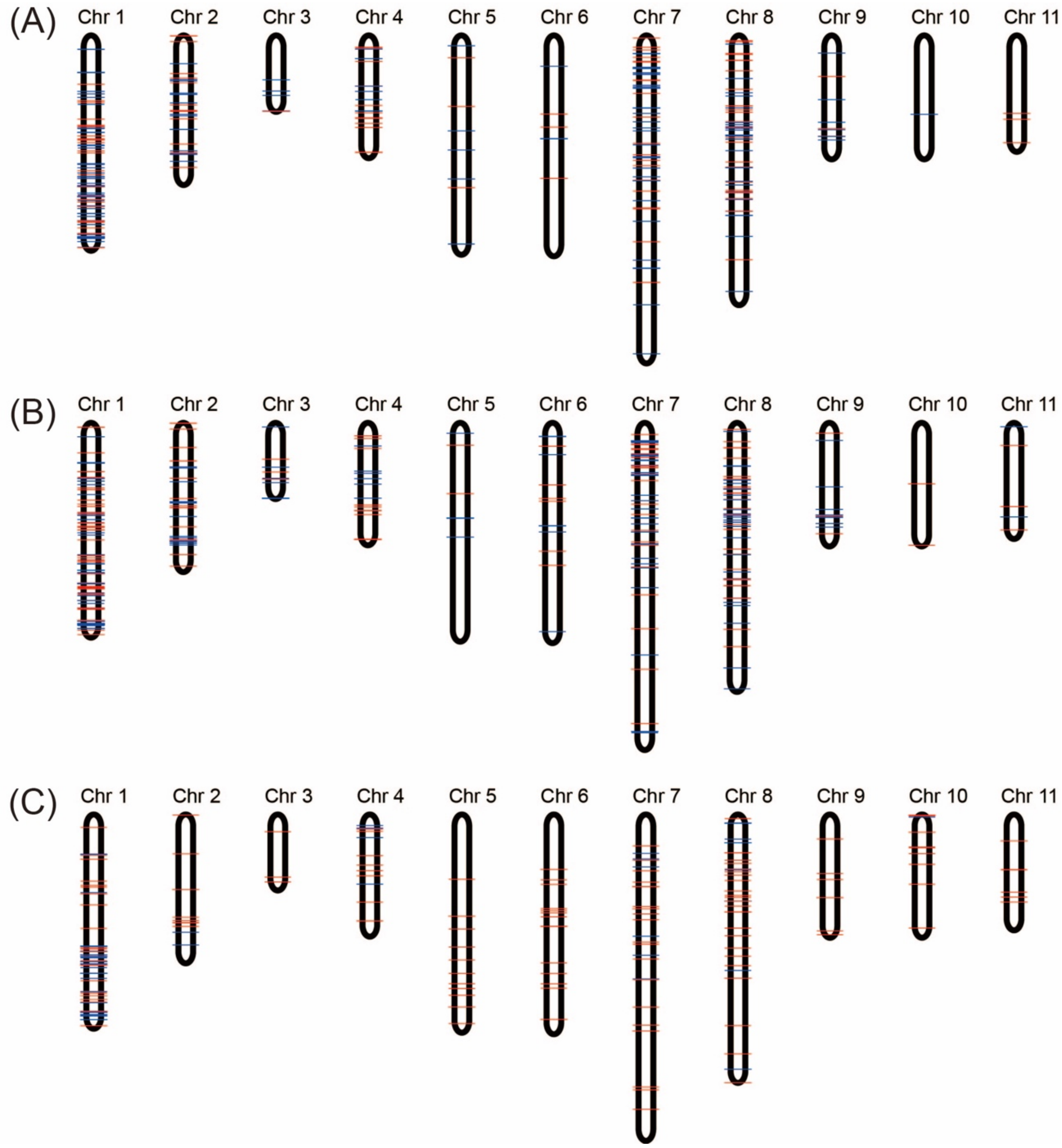

Figure S2. Distribution of DMRs in in cytosine contexts of (A) CpG (B) CHG (C) CHH. DMRs with higher methylation levels in high and low SPM groups were indicated by red and blue, respectively.

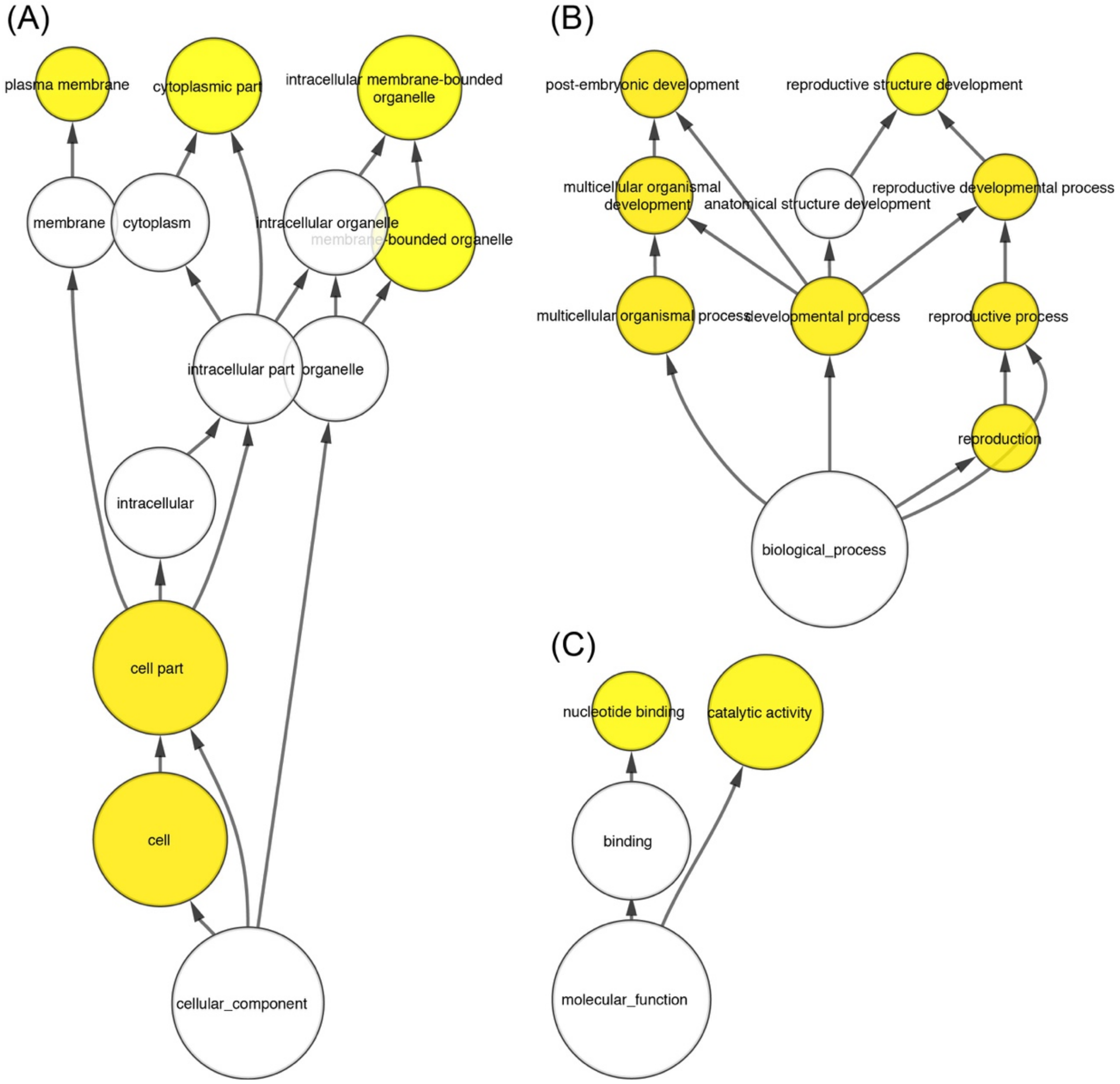

Figure S3. GO enrichment analysis among 211 genes proximal to genic DMRs. GO enrichment was analyzed using BiNGO plugin in Cytoscape software [25].

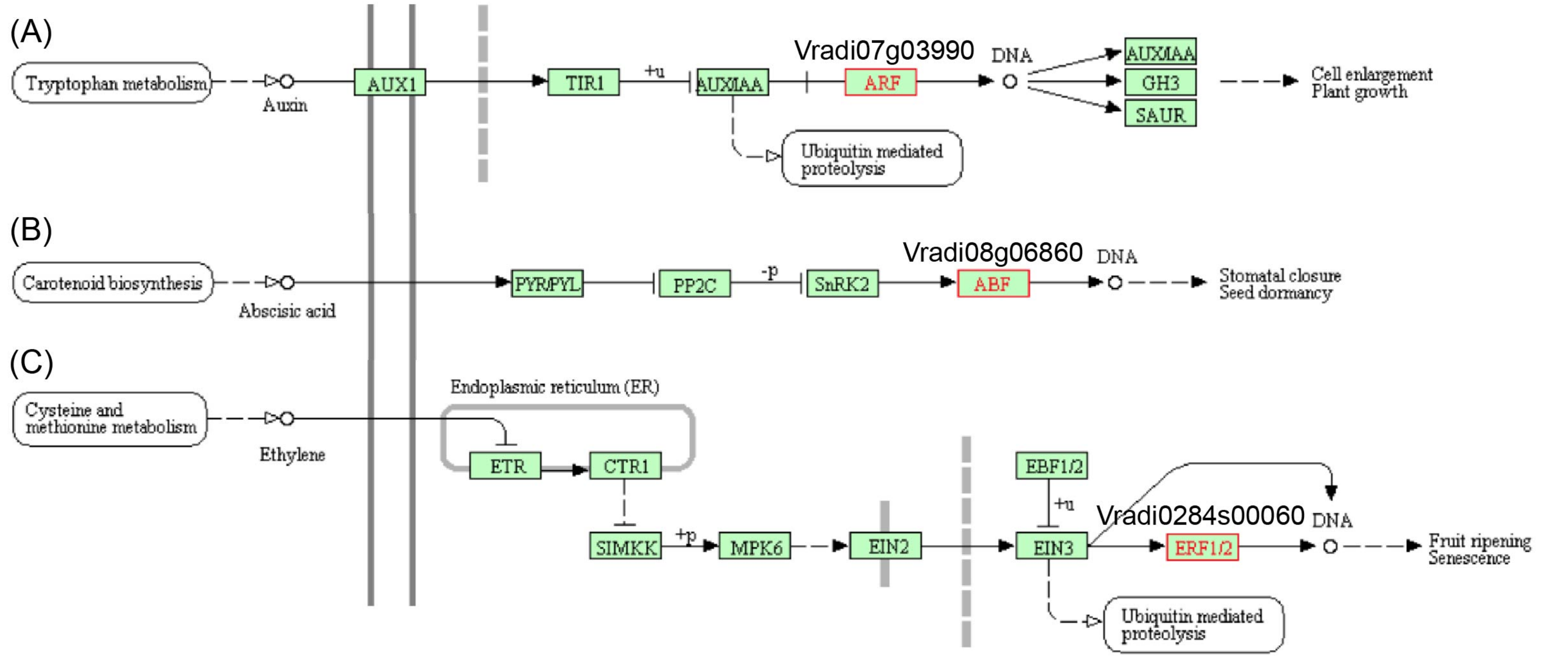

Figure S4. Transcription factors involved in hormone signaling pathways.

Arabidopsis orthologous genes of (A) Vradi07g03990, (B) Vradi08g06860 and (C) Vradi0284s00060 were mapped against auxin, abscisic acid and ethylene-related signal transduction, respectively, in KEGG pathway database [13].

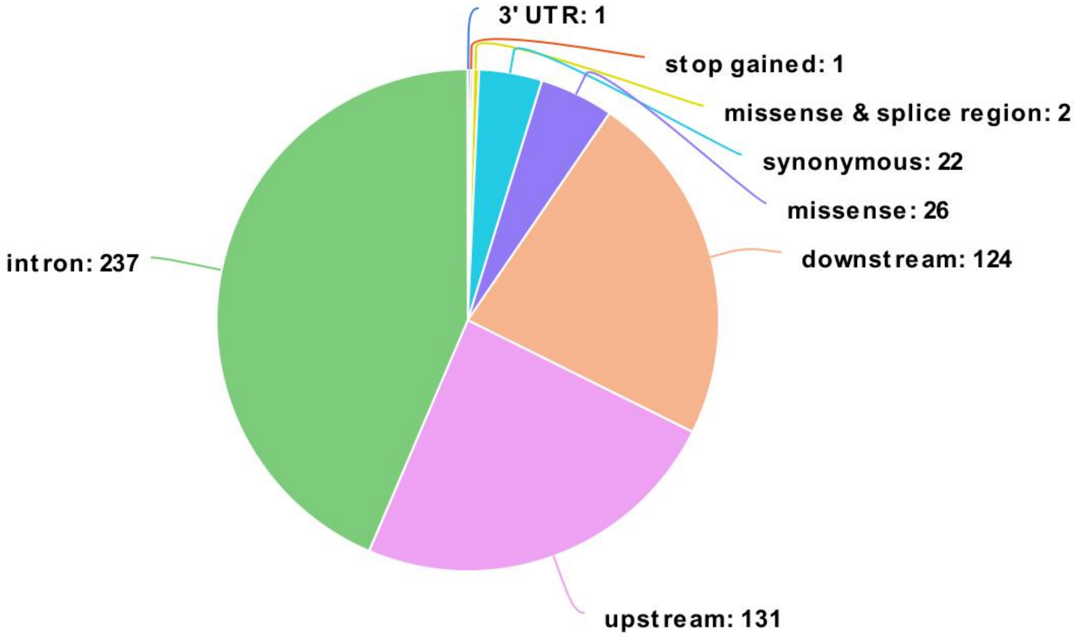

Figure S5. impact of SNPs located within 2 kbp upstream and downstream regions of 20 genes proximal to pure DMRs.
